# Both Gut Microbiota and Differentially Expressed Proteins Are Relevant to the Development of Obesity

**DOI:** 10.1155/2020/5376108

**Published:** 2020-09-24

**Authors:** Yuchuan Li, Qiuxia Liu, Chunting Peng, Bing Ruan

**Affiliations:** The First Affiliated Hospital, State Key Laboratory for Diagnosis and Treatment of Infectious Disease, College of Medicine, Zhejiang University, Hangzhou, Zhejiang Province, China

## Abstract

Although the role of the gut microbiota in obesity has recently received considerable attention, the exact mechanism is unclear. This study was aimed at investigating the profiles of bacterial communities in fecal samples and differentially expressed proteins (DEPs) in the peripheral blood in mice fed a high-fat diet (HFD) and standard diet (SD) and at providing new insights into the pathogenesis of obesity. The profiles of bacterial communities in fecal samples and DEPs in the peripheral blood were characterized in mice fed HFD and SD, respectively. The levels of 3 DEPs increased in HFD mice. The alpha diversity was significantly lower after 4 and 12 weeks in HFD mice. The beta diversity was higher after 4, 8, and 12 weeks in HFD mice. A total of 16 gut bacterial clades were significantly different with the linear discriminant analysis (LDA) score higher than 4 over time. The relative abundance levels of Proteobacteria and Deferribacteres were higher, while those of Bacteroidetes and Firmicutes were lower in HFD mice at the phylum level. The relative abundance of Desulfovibrionaceae and Rikenellaceae increased in HFD mice at the family level. The relative abundance of the Bacteroidetes_S24-7_group and Lachnospiraceae was lower in HFD mice. The gut microbiota had a significant correlation with serum lipid indexes and expression of DEPs at the phylum and family levels. The changes in the gut microbiota of HFD mice and their associations with the levels of inflammatory proteins could be one of the major etiological mechanisms underlying obesity.

## 1. Introduction

Obesity is a serious concern worldwide. Estimates show that the global prevalence of obesity will reach 18% in men and surpass 21% in women by 2025 [1]. According to the report of the global adult weight survey, China has surpassed the United States to become the country with the most obese individuals across the world. Numerous studies demonstrated an association of obesity with the relative abundance of two dominant bacterial divisions, including Bacteroidetes and Firmicutes [2–4]. Obesity, as a feature of metabolic abnormalities, has been linked with changes in the gut microbiota. It affects the energy storage and the metabolism of short-chain fatty acids and lipopolysaccharide (LPS) [4–6]. Meanwhile, the gut microbiota is an important environmental factor that affects diet-induced obesity [7]. In this study, diet-induced obesity models were used in mice to explore the mechanisms linking gut dysbiosis with obesity. However, the gut is a dynamic ecosystem. Previous studies focused on the alteration of the gut microbiota induced by high-fat diet (HFD) at experiment endpoints [8, 9]. However, a few studies specifically examined the change in the composition of the gut microbiota in response to HFD over time.

Accumulating studies have shown that diet-induced obesity is associated with chronic low-grade inflammation [10]. The commensal enteric bacteria can trigger a low-grade response by increasing the production of LPS [6, 10]. The amounts of proinflammatory cytokines and biomarkers of inflammation increase in diet-induced obese mice [11, 12]. However, the mechanisms underlying obesity-associated inflammation are not fully understood. Low-grade, systemic, and chronic inflammation induced by the diet-disrupted gut microbiota composition has been suggested as a primary pathological condition underlying the development of obesity [13]. Thus, it is essential to determine both the profiles of the gut microbiota and changes in cytokine levels in obesity.

Therefore, the dynamic changes in the gut microbiota were monitored and the levels of differentially expressed proteins (DEPs) were tested in a mouse model given HFD or standard diet (SD) for 12 weeks. Also, the associations of the gut microbiota at the phylum and family levels with serum lipid and DEP levels were analyzed to explain the response of mice to HFD-induced changes in the gut microbiota.

## 2. Materials and Methods

### 2.1. Animals

Wild-type (WT C57BL/6J, male, aged about 8 weeks, weighing about 20–23 g, specific pathogen-free (SPF)) mice were purchased from Huafukang Co. (Beijing, China) and bred in the Zhejiang University facility under a 12 : 12 h light/dark cycle. They were randomly divided into 2 groups (*n* = 20 each) fed HFD (45% kcal from fat, MD12032, Medicine Professional for Lab Animal Diets) and SD (10% kcal from fat, MD12031, Medicine Professional for Lab Animal Diets), respectively, for 12 weeks after 1 week of acclimatization. Food and water were supplied *ad libitum*. WT C57BL/6J mice were fed HFD for 12 weeks to establish a mouse obesity model using the Lee index. Weights were measured at 1-week intervals. The Lee index was calculated for12 weeks. Fecal samples were collected every 4 weeks and frozen at –80°C until use. All mice were sacrificed after 12 weeks of feeding. Whole blood was obtained from mouse eyeballs in an anticoagulant-free test tube, centrifuged to collect serum, and immediately stored at –80°C. All protocols and experiments were approved by the Institutional Animal Care and Use Committee of Zhejiang University (no. ZJU01007Y).

### 2.2. Measurement of Serum Lipid Levels

The plasma lipid levels were measured in 15 HFD and 8 SD mice after 12 weeks. Total cholesterol (TCHO), triglyceride (TG), high-density lipoprotein (HDL), and low-density lipoprotein (LDL) levels in blood samples were measured using specific kits (Nanjing Jiancheng Bioengineering Institute, China) according to the manufacturer's instructions.

### 2.3. Profiling of Serum DEPs

The expression of DEPs was detected using an AAM-CYT-G1000 Antibody Protein Array Kit (Ray Biotech, China). Fold change was used in the screening of DEPs (*P* < 0.001, fold change more than 1.2 and fluorescent values more than 150). All experiments were performed according to the manufacturer's protocol. First, after blocking, the chips were incubated at 4°C overnight with 100 *μ*L of serum samples. Then, the chips were thoroughly washed and incubated for 2 h with biotinylated antibodies at room temperature. Finally, the chips were washed again, followed by the addition of 70 *μ*L of labeled streptavidin to each well and incubation for 1 h in the dark at room temperature. After additional washes, fluorescence signals were visualized using an InnoScan 300 Microarray Scanner (RayBiotech, Norcross, GA, USA), and the data were analyzed using the AAM-CYT-G3 and AAM-CYT-G4 software.

### 2.4. Intestinal Microbiota Analysis

A total of 73 fecal samples from 24 mice were collected every 4 weeks during the dietary interventions (10, 11, 12, and 10 samples at 0, 4, 8, and 12 weeks in the HFD group, respectively; 11, 9, 12, and 9 samples at 0, 4, 8, and 12 weeks in the SD group, respectively). Total DNA was extracted from each 0.5 g fecal sample using a QIAamp Fast DNA Stool Mini Kit (Qiagen, German) following the manufacturer's protocol. Then, 16S rDNA sequencing was performed on an Illumina platform at Novogene Bioinformatics Technology Co. The microbiota composition of the samples was established by amplicon sequencing of the V3 and V4 regions of the 16S rRNA gene on an Illumina HiSeq2500 PE250. The sequences of primers were as follows: 341F-CCTAYGGGRBGCASCAG and 806R-GGACTACNNGGGTATCTAAT. All polymerase chain reactions (PCRs) were operated using Phusion High-Fidelity PCR Master Mix (New England Biolabs, USA). Raw tags were multiplexed and filtered using QIIME (V1.7.0, http://qiime.org/scripts/split_libraries_fastq.html) to obtain high-quality clean reads. Operational taxonomic units (OTUs) were clustered using UPARSE (V7.0.1001, http://drive5.com/uparse/) at a similarity level of 97%. The SSuRNA (http://www.arb-silva.de/) was used to annotate the representative OTU sequences and obtain the taxonomic information of each OTU. Alpha and beta diversity (unweighted UniFrac) analyses were performed using QIIME (version 1.7.0). The LDA Effect Size (LEfSe) analysis was performed using the LEfSe software to analyze species with significant differences in abundance between the two groups.

### 2.5. Statistical Analysis

All data were analyzed with the GraphPad Prism software 7.0 and shown as mean ± standard deviation. The Student *t*-test was performed for comparisons, with *P* < 0.05 considered statistically significant. The Spearman correlation analysis was carried out as the statistical method for two parameters; ^∗^*P* < 0.05, ^∗∗^*P* < 0.01, and ^∗∗∗^*P* < 0.001 represented the degree of correlation.

## 3. Results

### 3.1. Changes in Weight and Serum Lipid Levels in the Development of Obesity

**Figure 1:**
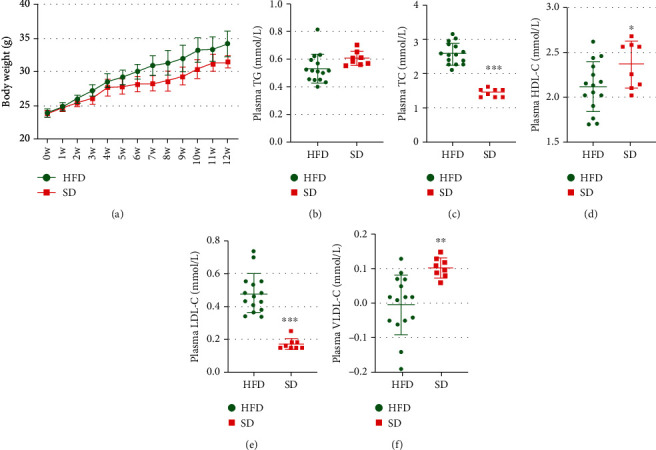
Weights and serum lipid levels in the two groups of mice: (a) comparison of weights; (b) levels of plasma TG; (c) levels of plasma TC; (d) levels of plasma HDL; (e) levels of plasma LDL; (f) levels of plasma VLDL. Levels of serum lipid indexes were determined at the end of the study. Data were expressed as mean ± SD. ^∗∗∗^*P* < 0.001, ^∗∗^*P* < 0.01, and ^∗^*P* < 0.05, by unpaired *t*-test (HFD, *n* = 15; SD, *n* = 8).

The weights were significantly higher in HFD mice compared with SD mice (*P* < 0.0001) (Figure 1(a)). However, the serum concentration of TG had no significant difference in HFD mice compared with SD mice (*P* > 0.05) (Figure 1(b)). The serum concentrations of TCHO significantly increased in HFD mice compared with SD mice (*P* < 0.0001) (Figure 1(c)). The HDL level was lower in HFD mice compared with SD mice (*P* < 0.05) (Figure 1(d)). The LDL level significantly increased in HFD mice compared with SD mice (*P* < 0.0001) (Figure 1(e)). The very low-density lipoprotein (VLDL) level was higher in SD mice compared with HFD mice (*P* < 0.01) (Figure 1(f)). These findings indicated that HFD alters serum lipoprotein levels in mice.

### 3.2. HFD Regulates the Inflammatory Response

**Figure 2:**
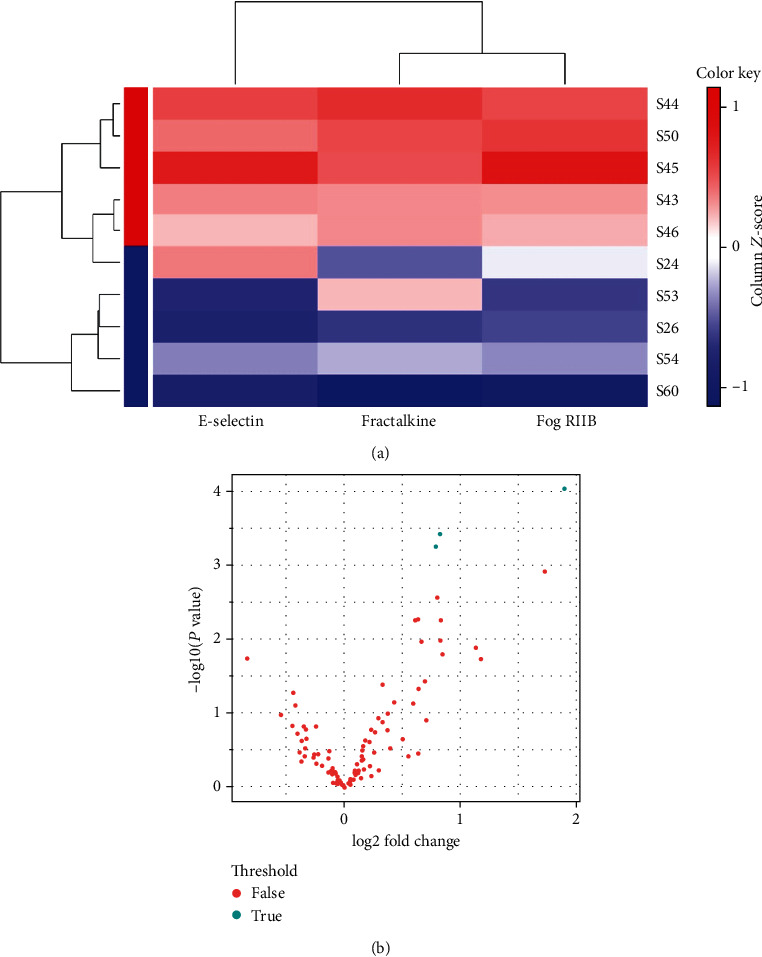
(a) Heat map clustering of 3 significant DEPs between the two groups. Red dots represent the HFD group. Blue dots represent the SD group. (b) Volcano plot of 96 cytokines in HFD and SD mice. Blue dots represent 3 significant DEPs (*P* < 0.001) (*n* = 5 in each group).

A total of 96 inflammation cytokines were examined at the protein level using an antibody protein array. Among them, 3 DEPs were found to be significantly different between HFD and SD mice (*P* < 0.001) (Figures 2(a) and 2(b)). Accurate testing data of the 3 DEPs are listed in Supplementary Table 1. The results were presented using a clustering heat map and a volcano plot (*P* < 0.001). Fractalkine, E-selectin, and FcgRIIB were significantly upregulated (*P* < 0.001).

Among 96 inflammation cytokines, 13 DEPs were found to be significantly different between HFD and SD mice (*P* < 0.05) (Supplementary Figs. 1a and 1b). Accurate testing data of the 13 DEPs are listed in Supplementary Table 2. The results were presented using a clustering heat map and a volcano plot (*P* < 0.05) (Supplementary Figs. 1c and 1d).

### 3.3. Profile of the Gut Microbiota in Response to Dietary Interventions

**Figure 3:**
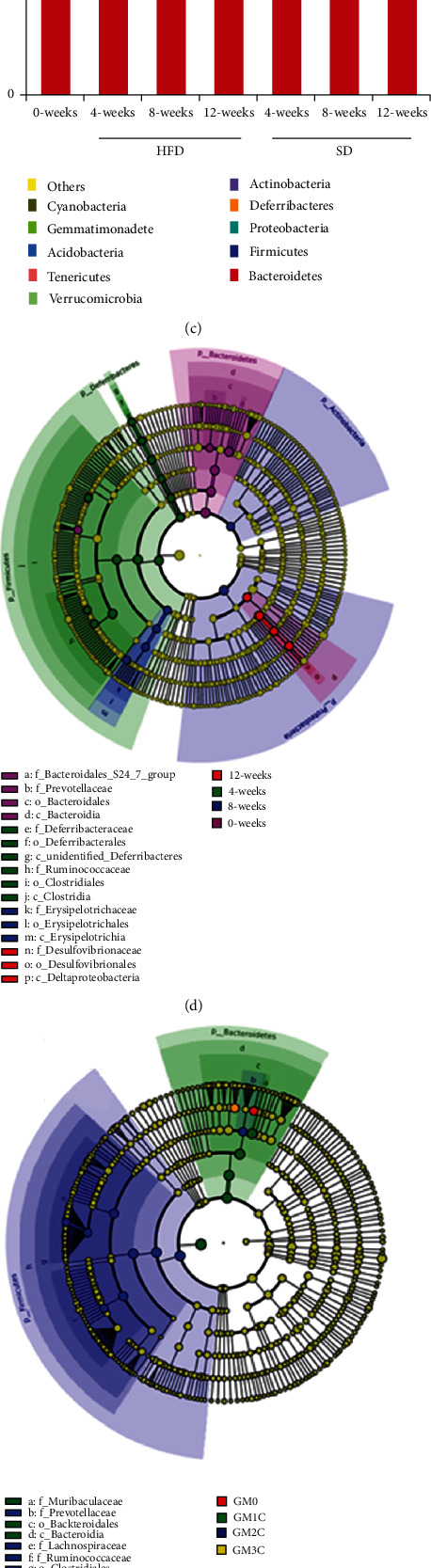
Gut microbial changes at four different time points. (a) Histograms of *α*-diversity (as assessed by the Shannon index) based on OTUs in HFD and SD mice. (b) Beta diversity was assessed by PCoA based on the OTUs of separate groups. (c) Relative abundance levels of the top 10 phyla between the two groups. (d) LEfSe analysis illustrated significant differences in the gut microbiota. The cladogram represents the taxonomic level of the phylum to the genus from inside to outside. The diameter of the circle is proportionated to each taxon's mean relative abundance. (e, f) Cladograms corresponding to the SD and HFD groups, respectively.

High-throughput sequencing of the V3 and V4 regions of the bacterial 16S rRNA gene was performed. The alpha diversity of the microbial communities was measured using the Shannon index (Figure 3(a)). The alpha diversity was significantly lower (*P* < 0.05) after 4 and 12 weeks in HFD mice compared with SD mice; however, no significant change was found after 8 weeks. The beta diversity was presented with UniFrac principal coordinate analysis (UniFrac-PCoA) based on OTU abundance during the development of obesity over time. The beta diversity was significantly higher (*P* < 0.05) after 4, 8, and 12 weeks in HFD mice compared with SD mice (Figure 3(b)). The relative abundance levels of the top 10 phyla showed dissimilarities in the fecal composition between the two groups (Figure 3(c)). Further, 16 gut bacterial clades were detected by LEfSe analysis showing significant differences with LDA scores higher than 4. The results were presented using cladograms (Figures 3(d)–3(f)).

### 3.4. Different Gut Microbial Communities at the Phylum and Family Levels

**Figure 4:**
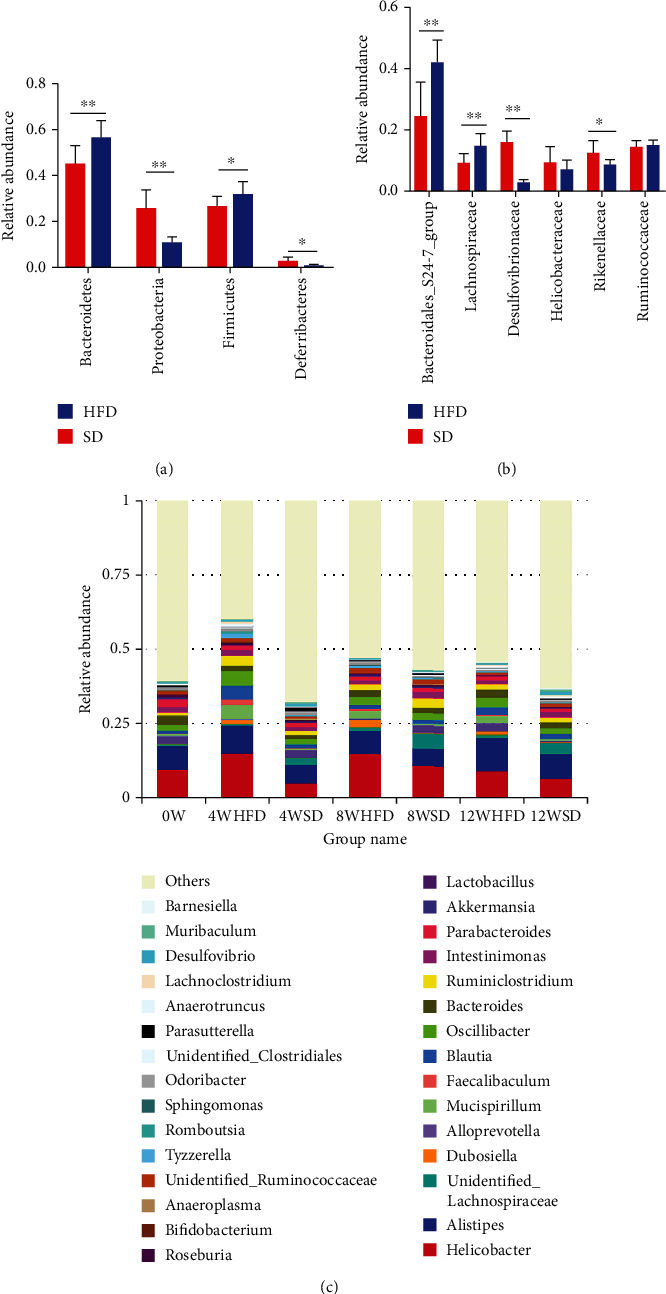
Different gut microbial communities between HFD and SD mice at the phylum and family levels. (a, b) Relative abundance levels of top four phyla and top six families are shown. (c) Top 30 differential genera between the SD and HFD groups. ^∗∗^*P* < 0.01 and ^∗^*P* < 0.05 using the unpaired *t*-test.

The data of the top four phyla and top six families in fecal samples collected after 12 weeks were used to explore the different microbial communities between HFD and SD mice. The relative abundance levels of Proteobacteria and Deferribacteres were higher (*P* < 0.05) in HFD mice at the phylum level. Additionally, significantly lower abundance levels of Bacteroidetes and Firmicutes were observed in HFD mice compared with SD mice (*P* < 0.05) (Figure 4(a)). The downstream analysis at the family level suggested that the relative abundance levels of Desulfovibrionaceae and Rikenellaceae significantly increased in HFD mice (*P* < 0.05). Inversely, the relative abundance levels of the Bacteroidetes_S24-7_group and Lachnospiraceae were lower in HFD mice compared with controls (*P* < 0.05) (Figure 4(b)). At the genus level, the top 30 differential genera detected between the SD and HFD groups included *Akkermansia*, *Phascolarctobacterium*, *Faecalibacterium*, and *Succinivibrio* (Figure 4(c)).

### 3.5. Relationships between the Gut Microbiota and Serum Lipid Indexes

**Figure 5:**
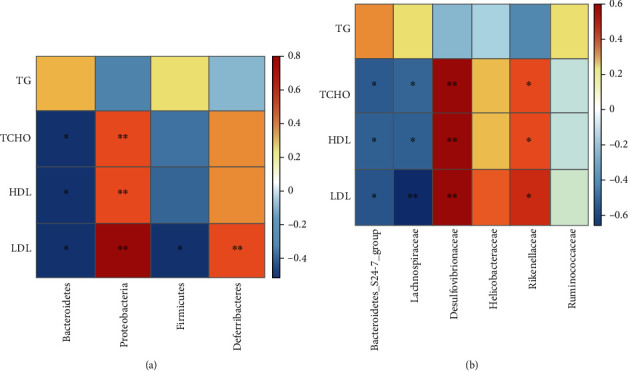
Correlations between the gut microbiota and serum lipid index. (a, b) Heat map of correlations between serum lipids and the relative abundance of the gut microbiota at the phylum and family levels. Spearman correlation coefficients are represented by color from blue (negative correlation) to red (positive correlation) from -0.5 to 0.5.

Next, the Spearman correlation analysis was performed to evaluate the association between gut microbiota alterations at the phylum and family levels and serum lipid indexes. The data were presented as a heat map. Significant correlations were noted as ^∗^*P* < 0.05 and ^∗∗^*P* < 0.01. At the phylum level, the relative abundance of Proteobacteria significantly positively correlated with the levels of TCHO, HDL, and LDL. The relative abundance of Deferribacteres significantly positively correlated with the level of LDL. Significantly negative relationships were observed between the relative abundance of Bacteroidetes and the levels of TCHO, HDL, and LDL. The relative abundance of *Firmicutes* was negatively correlated with the level of LDL (Figure 5(a)). At the family level, the levels of TCHO, HDL, and LDL positively correlated with the relative abundance levels of Desulfovibrionaceae and Rikenellaceae and negatively correlated with the relative abundance levels of the Bacteroidetes_S24-7_group and Lachnospiraceae (Figure 5(b)).

## 4. Discussion

In this study, diet-induced obesity mouse models were used to explore the underlying mechanism of obesity by dynamically analyzing the differences and correlations of the gut microbiota with DEPs in HFD and SD mice. The results showed that the characteristics of the gut microbiota changed substantially in obesity, with significant differences between the two groups. A total of 16 gut bacterial clades showed significant differences. Furthermore, the study demonstrated the correlations between the gut microbiota and DEPs (Supplementary Figs. 1a–1d, Supplementary Table 2). The results (Supplementary Figs. 1c and 1d) revealed that Bacteroidetes and the Bacteroidetes_S24-7_group showed negative correlations with the expression of DEPs. Inversely, positive correlations were observed between Proteobacteria, Deferribacteres, Desulfovibrionaceae, and Rikenellaceae and the expression of DEPs. Since the gut microbiota and DEPs are affected by HFD, future studies will determine whether one or both factors could change the likelihood of developing obesity.

A previous study showed that diet-induced weight loss in humans led to a reduction in plasma eotaxin levels. Fractalkine (CX3CL1) plays a pivotal role in the recruitment, infiltration, and proinflammatory polarization of leukocytes and microglial cells [14]. CX3CL1 is rapidly induced after the introduction of an HFD, and its inhibition impaired the induction of obese and glucose intolerance phenotypes [15]. Intercellular adhesion molecule 1 (ICAM-1) and selectins on the endothelium (E-selectin) are involved in the attachment of monocytes and lymphocytes to endothelial cells [16]. The elevated levels of ICAM-1 and E-selectin were found in children with obesity and adolescents [17]. These results suggested that obesity is associated with a low-grade systemic and chronic inflammatory condition [18]. Mice have four different FcgR types, including FcgRI, FcgRIIB, FcgRIII, and FcgRIV, with FcgRIIB being the only inhibitory receptor, while the others induce immune responses [19]. A study indicated that FcgRIIB suppresses atherosclerosis in mice [20]. As shown above, FcgRIIB was significantly upregulated, indicating that this might be a reparative mechanism reducing inflammation in HFD-fed animals.

This study found that the Shannon index was lower in HFD mice after 4 and 12 weeks; however, no significant change was found after 8 weeks. A previous study reported lower bacterial diversity in obese mice compared with lean individuals [21]. Beta diversity was significantly higher in HFD mice. The LEfSe analysis showed that the composition of the gut microbiota changed significantly over time. The present study reported significant changes in the prevalence of the class Clostridia and the order Bacteroidales in obesity. A previous study showed that the prevalence of the class Clostridia in the ileum decreased and the prevalence of the order Bacteroidales increased in HFD mice [22]. At the genus level, several were altered by HFD, including *Akkermansia*, *Phascolarctobacterium*, *Faecalibacterium*, and *Succinivibrio*. *Akkermansia* spp. have important roles in improving both glucose homeostasis and weight loss [23]. Meanwhile, Phascolarctobacterium is a producer of short-chain fatty acids (SCFAs), including butyrate, acetate, and propionate, which positively correlate with weight loss, and *Faecalibacterium prausnitzii* also produces butyrate [24, 25]. It is also known that *Succinivibrio* helps regulate the energy balance [26]. Although the link between the gut microbiota and obesity in HFD mice has been well studied, the demonstration of the causality between the constituents of the microbiota and obesity remains a huge challenge in this field. This suggests that further research is needed to determine the action of specific species on the development of obesity.

In a polysaccharide-rich diet-fed obese mouse model, a 50% reduction in the abundance of Bacteroidetes and a proportional increase in Firmicutes were observed [27]. Duranti et al. also showed that obesity is associated with changes in the relative abundance levels of the two dominant bacterial divisions Bacteroidetes and Firmicutes [3]. The relative abundance of Bacteroidetes increased as individuals with obesity lost weight. In this study, the prevalence of both Bacteroidetes and Firmicutes was significantly lower in HFD mice compared with control animals. The contradictory results might be related to feeding time, environment, and sample size. On switching from an SD to an HFD, WT mice become obese, accompanied by an increase in Proteobacteria [28]. Higher proportions of the phylum Deferribacteres were observed in two previous studies [29, 30]. The prevalence of the family Desulfovibrionaceae significantly increased in HFD mice. This was supported by the findings that one phylotype in the family Desulfovibrionaceae is more observed in WT/HFD obese mice [31]. The sulfate-reducing bacteria in the family Desulfovibrionaceae as potential endotoxin producers were associated with the development of obesity in the mouse model. The prevalence of the family Lachnospiraceae increased during long-term HFD consumption with an increase in the inflammatory status [32]. However, the prevalence of Lachnospiraceae was significantly lower in HFD mice. Truax et al. reported that a protective role of NLRP12 in obesity is associated with the prevalence of Lachnospiraceae and their metabolites [33]. The findings of Truax et al. were consistent with the present data. These findings implied that dysbiosis of the microbiota might contribute to the development of obesity via the underlying pathological mechanisms.

Although the present study confirmed the associations of the gut microbiota and expression of DEPs with obesity, it had two obvious limitations. First, it could not determine the causal relationship between the gut microbiota, expression of DEPs, and obesity. Secondly, human feces and serum should be collected to study the exact mechanism underlying obesity in humans. In a word, significant differences in the gut microbiota and the expression of DEPs were found in obesity, and the correlation between them was analyzed for providing new insight into weight management. Finally, although LPS represents an important microbial metabolite related to the inflammatory process and obesity, it was not measured over time in the current study.

## 5. Conclusions

This study showed that HFD induces changes in the gut microbiota through amplifying systematic proinflammatory responses involved in the mechanisms underlying obesity. Interventions with the gut microbiota may reduce HFD-induced systemic inflammation to protect against obesity. Further studies are needed to explore the role of DEPs in the cause-and-effect relationship in HFD-induced obesity.

## Data Availability

The datasets used and/or analyzed during the current study are available from the corresponding author on reasonable request.

## Supplementary Materials

Supplementary 1 Supplementary Figure 1: (a) heat map clustering of 13 significant DEPs between the two groups. Red dots represent the HFD group. Blue dots represent the SD group. (b) Volcano plot of 96 cytokines in HFD and SD mice. Blue dots represent 13 significant DEPs (*P* < 0.05) (*n* = 5 in each group). Relationship between the gut microbiota and the expression of DEPs. (c, d) Heat map of correlations between the expression of significant 13 DEPs and the relative abundance of the gut microbiota at the phylum and family levels. The Spearman correlation coefficients are represented by color from blue (negative correlation) to red (positive correlation) from -0.5 to 0.5.

Supplementary 2 Supplementary Table 1: accurate testing data of 3 DEPs (*P* value, log2 fold change, adjusted *P* value). Supplementary Table 2: accurate testing data of 13 DEPs (*P* value, log2 fold change, fluorescent mean values).
