# Supplementary material for: Both Gut Microbiota and Differentially Expressed Proteins Are Relevant to the Development of Obesity
Source: Biomed Res Int. 2020 Sep 24;2020:5376108. doi: 10.1155/2020/5376108 (PMC7533028; doi:10.1155/2020/5376108)
Supplement: Supplementary 1 — Supplementary Figure 1: (a) heat map clustering of 13 significant DEPs between the two groups. Red dots represent the HFD group. Blue dots represent the SD group. (b) Volcano plot of 96 cytokines in HFD and SD mice. Blue dots represent 13 significant DEPs (P < 0.05) (n = 5 in each group). Relationship between the gut microbiota and the expression of DEPs. (c, d) Heat map of correlations between the expression of significant 13 DEPs and the relative abundance of the gut microbiota at the phylum and family levels. The Spearman correlation coefficients are represented by color from blue (negative correlation) to red (positive correlation) from -0.5 to 0.5. [file 5376108.f1.docx]

Supplementary figure


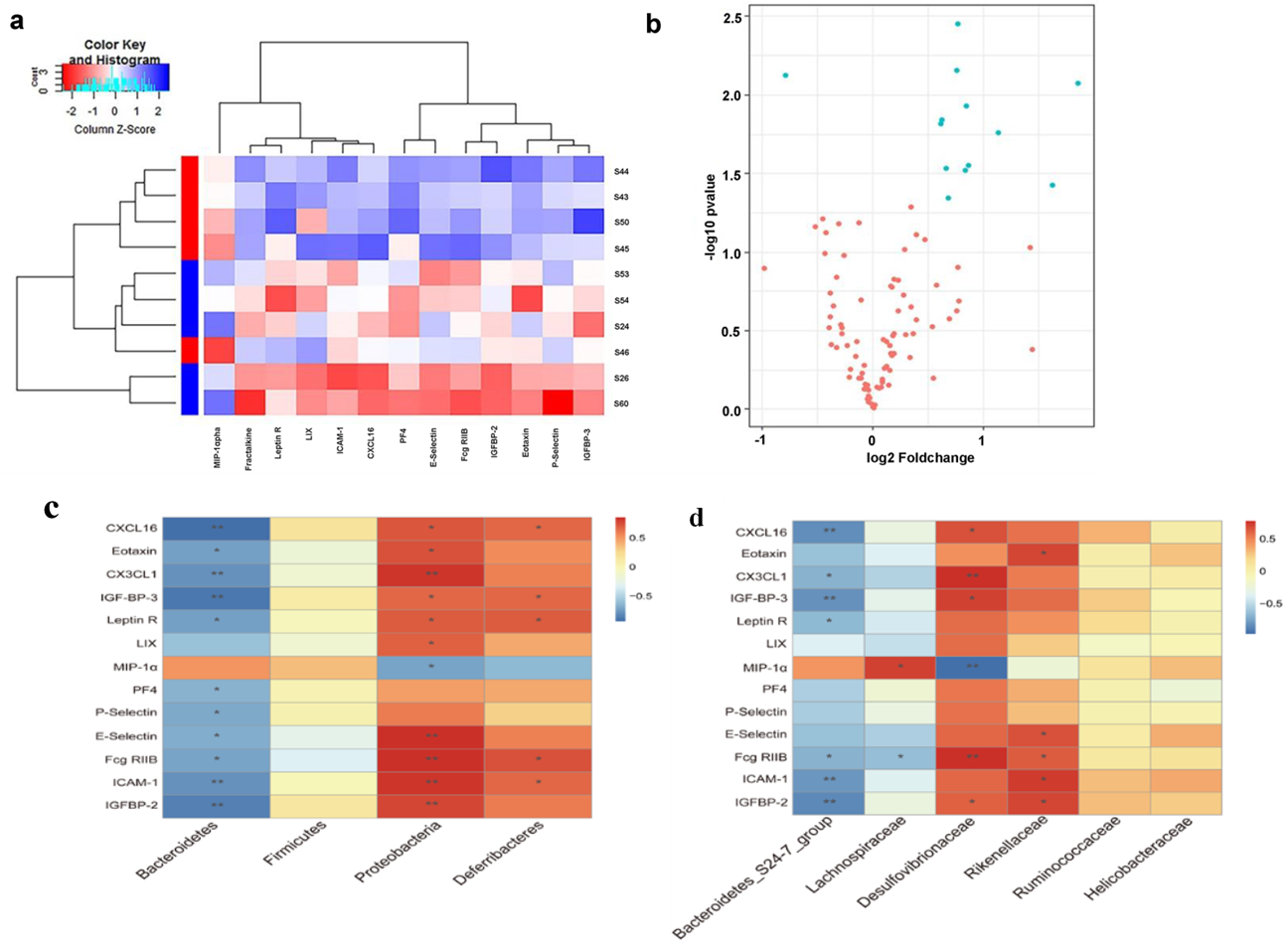


Figure 1. (a) Heatmap clustering of 13 significant DEPs between the two groups. Red dots represent the HFD group. Blue dots represent the SD group. (b) Volcano plot of 96 cytokines in HFD and SD mice. Blue dots represent 13 significant DEPs (*P* < 0.05) (*n* = 5 in each group). Relationship between gut microbiota and the expression of DEPs. (c and d) Heatmap of correlations between the expression of significant 13 DEPs and the relative abundance of the gut microbiota at the phylum and family levels. Spearman correlation coefficients are represented by color from blue (negative correlation) to red (positive correlation) from -0.5 to 0.5.
