# Supplementary material for: Both Gut Microbiota and Differentially Expressed Proteins Are Relevant to the Development of Obesity
Source: Biomed Res Int. 2020 Sep 24;2020:5376108. doi: 10.1155/2020/5376108 (PMC7533028; doi:10.1155/2020/5376108)
Supplement: Supplementary 2 — Supplementary Table 1: accurate testing data of 3 DEPs (P value, log2 fold change, adjusted P value). Supplementary Table 2: accurate testing data of 13 DEPs (P value, log2 fold change, fluorescent mean values). [file 5376108.f2.docx]

**Supplementary Table 1: Accurate testing data of 3 DEPs (p-value, log2Foldchange, adjust p-value )**

| DEPs (13) |  |  |  | HFD | HFD | HFD | HFD | HFD | SD | SD | SD | SD | SD |
| --- | --- | --- | --- | --- | --- | --- | --- | --- | --- | --- | --- | --- | --- |
| Value, the sample number | log2Foldchange | *P* value | Adj.*P.*value | S46 | S43 | S44 | S45 | S50 | S26 | S24 | S53 | S60 | S54 |
| Fcg RIIB | 3.7380 | 0.00009 | 0.00900 | 17069.85 | 18923.13 | 26620.74 | 41096.33 | 28864.45 | 6296.29 | 12149.40 | 5972.64 | 3462.03 | 8830.89 |
| Fractalkine | 1.7653 | 0.00038 | 0.01807 | 3572.85 | 3533.65 | 4406.66 | 4089.25 | 4109.50 | 2031.17 | 2209.06 | 3299.73 | 1412.12 | 2605.76 |
| E-Selectin | 1.73650 | 0.00056 | 0.01807 | 216200.8 | 235886.1 | 273578.1 | 314954.1 | 244367.8 | 121747.6 | 237764.7 | 125255.3 | 117354.4 | 159823.2 |
|  |  |  |  |  |  |  |  |  |  |  |  |  |  |

**Supplementary Table 2: Accurate testing data of 13 DEPs (p-value, log2Foldchange, fluorescent mean values)**

| DEPs (13) | HFD | SD |  |  | HFD | HFD | HFD | HFD | HFD | SD | SD | SD | SD | SD |
| --- | --- | --- | --- | --- | --- | --- | --- | --- | --- | --- | --- | --- | --- | --- |
| Value, the sample number | mean B | mean C | log2Foldchange | *P* value | S46 | S43 | S44 | S45 | S50 | S26 | S24 | S53 | S60 | S54 |
| CXCL16 | 9,945.53 | 3,236.33 | 1.62 | 0.04 | 5,286.24 | 8,327.26 | 7,157.58 | 18,231.26 | 10,725.30 | 1,283.54 | 2,849.91 | 5,465.10 | 1,488.63 | 5,094.45 |
| Eotaxin | 40,355.36 | 26,390.24 | 0.61 | 0.02 | 29,528.60 | 41,965.73 | 48,235.12 | 39,447.73 | 42,599.64 | 24,485.74 | 37,419.26 | 29,935.92 | 21,693.39 | 18,416.91 |
| Fractalkine （CX3CL1） | 3,942.38 | 2,311.57 | 0.77 | 0.00 | 3,572.85 | 3,533.65 | 4,406.66 | 4,089.25 | 4,109.50 | 2,031.17 | 2,209.06 | 3,299.73 | 1,412.12 | 2,605.76 |
| IGF-BP-3 | 81,505.66 | 45,858.99 | 0.83 | 0.03 | 58,061.80 | 66,548.07 | 97,463.50 | 67,918.06 | 117,536.88 | 45,722.36 | 35,426.21 | 58,480.57 | 37,821.60 | 51,844.19 |
| Leptin R (Ob- R ) | 5,086.23 | 2,831.36 | 0.85 | 0.01 | 4,791.49 | 5,963.52 | 4,513.91 | 3,497.95 | 6,664.29 | 2,581.42 | 3,113.10 | 3,166.92 | 3,324.95 | 1,970.39 |
| LIX | 8,665.79 | 4,783.46 | 0.86 | 0.03 | 9,523.22 | 9,595.90 | 8,393.13 | 11,428.53 | 4,388.16 | 3,310.86 | 7,435.38 | 5,383.67 | 3,731.20 | 4,056.17 |
| MIP-1-alpha (CCL3) | 329.83 | 571.42 | -0.79 | 0.01 | 220.30 | 418.29 | 399.11 | 283.25 | 328.21 | 483.53 | 691.00 | 554.82 | 697.47 | 430.29 |
| PF4 (platelet factor 4) | 130,778.46 | 85,243.98 | 0.62 | 0.01 | 106,880.46 | 148,303.68 | 140,821.74 | 99,067.51 | 158,818.90 | 88,724.64 | 76,536.34 | 113,150.95 | 70,571.45 | 77,236.52 |
| P-Selectin | 62,092.45 | 38,806.82 | 0.68 | 0.05 | 57,698.65 | 55,539.60 | 70,421.29 | 55,741.87 | 71,060.85 | 31,001.32 | 40,596.41 | 64,007.44 | 13,733.39 | 44,695.53 |
| E-Selectin | 256,997.41 | 152,389.05 | 0.75 | 0.01 | 216,200.84 | 235,886.12 | 273,578.11 | 314,954.16 | 244,367.83 | 121,747.57 | 237,764.70 | 125,255.33 | 117,354.39 | 159,823.24 |
| Fcg RIIB | 26,514.90 | 7,342.25 | 1.85 | 0.01 | 17,069.86 | 18,923.13 | 26,620.74 | 41,096.33 | 28,864.45 | 6,296.29 | 12,149.40 | 5,972.64 | 3,462.03 | 8,830.89 |
| ICAM-1 | 6,443.28 | 2,950.15 | 1.13 | 0.02 | 3,269.26 | 5,992.98 | 8,140.38 | 8,678.07 | 6,135.72 | 1,569.42 | 3,771.40 | 2,566.27 | 2,555.12 | 4,288.56 |
| IGFBP-2 | 22,464.86 | 14,229.95 | 0.66 | 0.03 | 16,101.22 | 20,036.58 | 30,735.37 | 25,097.94 | 20,353.21 | 10,390.92 | 15,085.32 | 16,846.19 | 10,404.28 | 18,423.06 |
